# Common polymorphisms in *CD44* gene and susceptibility to cancer: a systematic review and meta-analysis of 45 studies

**DOI:** 10.18632/oncotarget.12580

**Published:** 2016-10-12

**Authors:** Meng Zhang, Yangyang Wang, Tingting Fang, Yangke Cai, Yue Xu, Cunye Yan, Li Zhang, Chaozhao Liang

**Affiliations:** ^1^ Department of Urology, the First Affiliated Hospital of Anhui Medical University, Hefei, China; ^2^ Institute of Urology, Anhui Medical University, Hefei, China; ^3^ Department of Urology, The Second People's Hospital of Guangdong Province, Guangzhou, China

**Keywords:** CD44, polymorphism, cancer, meta-analysis, systematic review

## Abstract

CD44 is one of the commonly recognized stem cell markers, which plays a critical role in many cancer related cellular processes. Relationships between *CD44* polymorphisms and cancer risk have been widely investigated previously, whereas results derived from these studies were inconclusive and controversial. We conducted present meta-analysis aiming to explore the association between *CD44* polymorphisms and cancer risk. We calculated pooled odds ratios (ORs) corresponding with the 95% confidence intervals (CIs) to make the evaluation clear. Embase, Web of Science, PubMed and Cochrane Library databases were retrieved to identify all eligible publications. As a result, a total of 12 publications comprised 25,777 cases and 27,485 controls fulfilled the inclusion criteria. Nevertheless, the pooled analyses suggested that no significant association was uncovered between *CD44* (rs10836347, rs11821102, rs13347, rs1425802, rs353639, rs713330 and rs187115) polymorphisms with overall cancer risk. Subsequently, we conducted subgroup analysis for rs13347 polymorphism based on source of control, and we identified a significantly increased cancer risk for the population-based (P-B) group restricted to a recessive model (TT vs. TC+CC: OR = 2.030, 95%CI: 1.163-3.545, *P*Adjust < 0.001). In conclusion, our meta-analysis demonstrates that *CD44* polymorphisms may not represent risk factors for cancer. Future well-designed large-scale case-control studies are warranted to verify our findings.

## INTRODUCTION

Malignant tumors pose serious threats to human health and are currently among the top causes of death [1]. In this era of precision medicine, the identification of ideal biomarkers for diagnosis to optimize the prevention and treatment of malignant tumors has become a hotspot in both research and clinical practice.

CD44 was primarily demonstrated as a receptor for the hyaluronan and lymphocyte-homing receptor [2]. Recently, this multi-structural and multi-functional transmembrane glycoprotein has been demonstrated to play a pivotal part in evaluating prognosis for a variety of cancer types, such as bile duct cancer [3], colorectal cancer (CRC) [4] and breast cancer (BC) [5]. CD44 is expressed as different isoforms derived from alternative splicing of variant exons [6]. And common isoforms of CD44, which have been identified related to cancer metastasis, are the surface adhesion molecules. In 1990s, CD44v6 was widely-accepted to be the major variant isoform in rat carcinoma cells participated in the regulation of tumor metastasis [7]. Besides, CD44v6 also expressed in both premature and mature lung tissues and connected with epithelial stem cells [8].

Several recent studies have demonstrated that many polymorphisms in *CD44* were correlated with the risk of many cancers, including BC [9], gastric cancer (GC) [10] and CRC [11]. In Jiang *et al.*'s study [9], the authors identified that rs13347 CT + TT genotype increased individuals' susceptibility to BC relative to the most common CC genotype, particularly for estrogen receptor (ER) negative patients. Consistently, Wu *et al.* [11] verified these results in CRC. In addition, the functional assays demonstrated that rs13347 polymorphism C to T base change disrupted the binding site for mir-509-3p, thus, the transcriptional activity was increased, as well as the expression level of CD44. Later on, Tulsyan *et al.* [12] revealed that *CD44* rs353639 polymorphism potentially has a significant effect in BC patients' prognosis. Nevertheless, both rs13347 and rs353639 polymorphisms had no influence on BC risk. Noting these controversial and inconclusive results, we conducted the current meta-analysis in order to determine a more exact relationship between *CD44* polymorphisms and the risk of cancer.

## RESULTS

### Characteristics of the eligible studies

**Figure 1:**
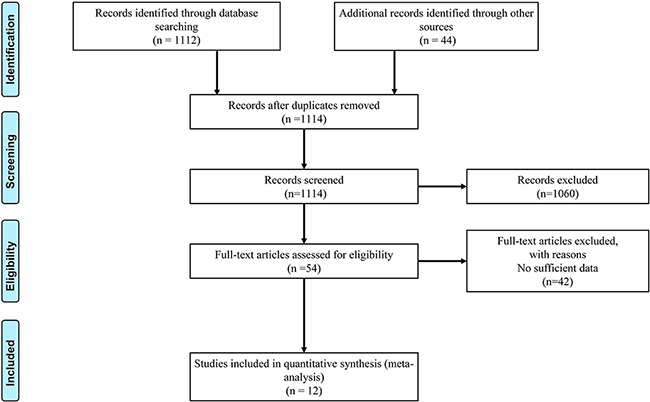
Flow diagram of included studies for the meta-analysis

A sum of 12 publications that met the inclusion criteria were enrolled in the quantitative synthesis (Figure 1 and Table 1) [9–20]. For rs10836347 polymorphism, we identified six studies encompassing 4,124 cases and 4,672 controls. The ethnicities of all these studies were Asian populations. For rs11821102 polymorphism, we enrolled seven qualified studies consisted of 4,399 cases and 4,947 controls. For rs13347 polymorphism, ten publications met the inclusion criteria, comprising 6,438 cases and 6,511 controls. Among the ten studies, seven studies were conducted in Asian populations and the others were in Caucasian populations. For rs1425802 polymorphism, we identified six qualified Asian studies including 3,453 cases and 3,961 controls. For rs187115 polymorphism, we identified six qualified studies comprising 2,326 cases and 2,315 controls. Among these six studies, four studies were conducted in Asian populations and the other two were in Caucasian populations. For rs353639 polymorphism, four qualified studies including 1,584 cases and 1,120 controls were enrolled. Three studies were performed in Caucasian populations, and one in Asian population. For rs713330 polymorphism, we identified six qualified Asian studies comprising 3,453 cases and 3,959 controls.

**Table 1:** Baseline Characteristics of studies included

SNP	First Author	Year	Ethnicity	Genotyping Method	Source of Control	Cancer Type	Case			Control			
							AA	AB	BB	AA	AB	BB	Y(HWE)
rs13347	Wu *et al.*	2015	Asian	RT-PCR	H-B	CRC	416	441	89	578	348	63	Y
C>T	Chou *et al.*	2014	Asian	RT-PCR	H-B	HCC	110	72	21	295	223	43	Y
	Wu *et al.*	2013	Asian	Mass Array	H-B	AML	163	196	62	254	171	36	Y
	Tulsyan *et al.*	2013	Caucasian	TaqMan	P-B	BC	191	60	7	178	57	6	Y
	Jiang *et al.*	2012	Asian	Mass Array	H-B	BC	813	850	190	1146	727	119	Y
	Xiao *et al.*	2013	Asian	Mass Array	P-B	BC	386	418	102	606	297	40	Y
	Chou *et al.*	2014	Asian	RT-PCR	H-B	BC	295	223	43	287	262	50	Y
	Sharma *et al.*	2013	Caucasian	TaqMan	H-B	BC	293	104	8	154	42	4	Y
	Yadav *et al.*	2015	Caucasian	TaqMan	P-B	GBC	378	201	31	162	80	8	Y
	Weng *et al.*	2015	Asian	TaqMan	H-B	UBC	143	117	15	138	111	26	Y
rs10836347	Wu *et al.*	2015	Asian	RT-PCR	H-B	CRC	821	120	5	851	129	9	Y
C>T	Chou *et al.*	2014	Asian	RT-PCR	H-B	HCC	180	23	0	487	69	5	Y
	Wu *et al.*	2013	Asian	Mass Array	H-B	AML	364	55	2	404	55	2	Y
	Jiang *et al.*	2012	Asian	Mass Array	H-B	BC	906	139	4	995	156	6	Y
	Xiao *et al.*	2013	Asian	Mass Array	P-B	NC	785	118	3	792	147	4	Y
	Chou *et al.*	2014	Asian	RT-PCR	H-B	OSCC	522	73	4	487	69	5	Y
rs11821102	Wu *et al.*	2015	Asian	RT-PCR	H-B	CRC	815	119	12	843	131	15	N
A>G	Chou *et al.*	2014	Asian	RT-PCR	H-B	HCC	173	29	1	481	75	5	Y
	Wu *et al.*	2013	Asian	Mass Array	H-B	AML	370	50	1	398	59	4	Y
	Jiang *et al.*	2012	Asian	Mass Array	H-B	BC	912	125	12	997	151	9	Y
	Xiao *et al.*	2013	Asian	Mass Array	P-B	NC	796	100	10	805	129	9	Y
	Chou *et al.*	2014	Asian	RT-PCR	H-B	OSCC	531	63	5	481	75	5	Y
	Weng *et al.*	2015	Asian	TaqMan	H-B	UBC	222	50	3	234	39	2	Y
rs1425802	Chou *et al.*	2014	Asian	RT-PCR	H-B	HCC	70	75	58	197	235	132	N
A>G	Wu *et al.*	2013	Asian	Mass Array	H-B	AML	126	204	91	122	248	91	Y
	Jiang *et al.*	2012	Asian	Mass Array	H-B	BC	316	513	220	353	563	241	Y
	Xiao *et al.*	2013	Asian	Mass Array	P-B	NC	270	450	186	299	442	202	Y
	Chou *et al.*	2014	Asian	RT-PCR	H-B	OSCC	197	249	153	194	235	132	N
	Weng *et al.*	2015	Asian	TaqMan	H-B	UBC	99	109	67	105	121	49	Y
rs187115	Liu *et al.*	2015	Asian	TaqMan	H-B	NSCLC	133	86	15	336	119	13	Y
A>G	Chou *et al.*	2014	Asian	RT-PCR	H-B	HCC	123	66	14	403	143	15	Y
	Sharma *et al.*	2013	Caucasian	ARMS-PCR	H-B	GBC	248	126	31	125	61	14	Y
	Chou *et al.*	2014	Asian	RT-PCR	H-B	OSCC	336	227	36	403	143	15	Y
	Yadav *et al.*	2015	Caucasian	TaqMan	P-B	GBC	353	178	79	150	70	30	N
	Weng *et al.*	2015	Asian	TaqMan	H-B	UBC	204	68	3	178	87	10	Y
rs713330	Chou *et al.*	2014	Asian	RT-PCR	H-B	HCC	167	36	0	467	86	8	Y
C>T	Wu *et al.*	2013	Asian	Mass Array	H-B	AML	341	74	6	371	87	3	Y
	Jiang *et al.*	2012	Asian	Mass Array	H-B	BC	865	172	12	950	194	13	Y
	Xiao *et al.*	2013	Asian	Mass Array	P-B	NC	732	164	10	751	180	12	Y
	Chou *et al.*	2014	Asian	RT-PCR	H-B	OSCC	507	88	4	467	86	8	Y
	Weng *et al.*	2015	Asian	TaqMan	H-B	UBC	223	49	3	231	42	3	Y
rs353639	Tulsyan *et al.*	2013	Caucasian	TaqMan	P-B	BC	158	89	11	150	89	13	Y
A>C	Sharma *et al.*	2013	Caucasian	TaqMan	H-B	GBC	253	130	22	120	68	12	Y
	Qiu *et al.*	2014	Asian	Mass Array	H-B	GC	211	90	10	310	100	8	Y
	Yadav *et al.*	2015	Caucasian	TaqMan	P-B	GBC	388	174	48	167	68	15	N

AML: Acute myeloid leukemia; BC: breast cancer; NC: Nasopharyngeal carcinoma; OSCC: Oral squamous cell carcinoma; GBC: Gallbladder cancer; GC: Gastric cancer; RT-PCR: reverse transcription-polymerase chain reaction; ARMS-PCR: Amplification Refractory Mutation System-Polymerase Chain Reaction; P-B: population-based; H-B: hospital-based; HWE: Hardy Weinberg Equilibrium; A: wild type; B: mutated type.

Table 1 summarized the demographic characteristics of these selected studies enrolled in present meta-analysis. As shown in Table 1, genotyping methods applied in these studies included MassArray, reverse transcription-polymerase chain reaction (RT-PCR), Amplification Refractory Mutation System-Polymerase Chain Reaction (ARMS-PCR) and TaqMan. In addition, there were five case-control studies whose genotype distributions in the control groups were not conformed to Hardy-Weinberg equilibrium (HWE) (Table 1) [11, 14–17]. For these studies, subgroup analyses by HWE status and sensitivity analyses were conducted to evaluate the potential effects of these studies on the overall pooled results.

### Pooled analysis

The association between *CD44* genetic polymorphisms and risk of cancer was shown in Table 2. No any statistically significant association was found between *CD44* polymorphisms (rs10836347, rs11821102, rs13347, rs1425802, rs353639, rs713330 and rs187115) and overall cancer risk in all the five genetic models (Table 2).

**Table 2:** The overall analyses of *CD44* polymorphisms and cancer risk

SNP	Comparison	Subgroup	N	*P*H	*P*Z	*P* (Adjust)	Random	Fixed
rs13347	T VS. C	Overall	10	0.000	**0.013**	0.455	1.272 (1.052-1.538)	1.426 (1.348-1.508)
C > T	T VS. C	Asian	7	0.000	**0.016**	0.560	1.328 (1.055-1.671)	1.462 (1.378-1.552)
	T VS. C	Caucasian	3	0.726	0.184	1.000	1.129 (0.942-1.353)	1.130 (0.944-1.353)
	T VS. C	MassArray	3	0.005	**0.000**	**0.000**	1.766 (1.454-2.144)	1.706 (1.581-1.842)
	T VS. C	RT-PCR	3	0.000	0.554	1.000	1.122 (0.766-1.643)	1.207 (1.091-1.335)
	T VS. C	TaqMan	4	0.348	0.618	1.000	1.038 (0.887-1.215)	1.038 (0.895-1.204)
	T VS. C	H-B	7	0.000	0.055	1.000	1.229 (0.996-1.516)	1.360 (1.276-1.449)
	T VS. C	P-B	3	0.000	0.216	1.000	1.366 (0.833-2.242)	1.686 (1.494-1.904)
	T VS. C	BC	5	0.000	0.105	1.000	1.302 (0.946-1.792)	1.478 (1.376-1.588)
	TC VS. CC	Overall	10	0.000	**0.018**	0.630	1.302 (1.046-1.622)	1.476 (1.370-1.591)
	TC VS. CC	Asian	7	0.000	**0.018**	0.630	1.378 (1.058-1.796)	1.536 (1.419-1.663)
	TC VS. CC	Caucasian	3	0.619	0.347	1.000	1.107 (0.893-1.372)	1.108 (0.895-1.373)
	TC VS. CC	MassArray	3	0.053	**0.000**	**0.000**	1.857 (1.528-2.257)	1.806 (1.629-2.002)
	TC VS. CC	RT-PCR	3	0.000	0.754	1.000	1.091 (0.634-1.877)	1.236 (1.080-1.415)
	TC VS. CC	TaqMan	4	0.772	0.394	1.000	1.082 (0.901-1.299)	1.083 (0.902-1.300)
	TC VS. CC	H-B	7	0.000	0.058	1.000	1.274 (0.992-1.637)	1.423 (1.307-1.550)
	TC VS. CC	P-B	3	0.000	0.300	1.000	1.351 (0.765-2.388)	1.662 (1.424-1.939)
	TC VS. CC	BC	5	0.000	0.116	1.000	1.332 (0.932-1.903)	1.523 (1.386-1.674)
	TC+TT VS. CC	Overall	10	0.000	**0.016**	0.560	1.335 (1.055-1.691)	1.537 (1.431-1.650)
	TC+TT VS. CC	Asian	7	0.000	**0.016**	0.560	1.421 (1.068-1.892)	1.602 (1.486-1.728)
	TC+TT VS. CC	Caucasian	3	0.672	0.247	1.000	1.129 (0.918-1.389)	1.130 (0.919-1.389)
	TC+TT VS. CC	MassArray	3	0.015	**0.000**	**0.000**	2.003 (1.603-2.502)	1.929 (1.749-2.128)
	TC+TT VS. CC	RT-PCR	3	0.000	0.666	1.000	1.125 (0.658-1.922)	1.262 (1.110-1.436)
	TC+TT VS. CC	TaqMan	4	0.631	0.445	1.000	1.070 (0.897-1.276)	1.071 (0.898-1.276)
	TC+TT VS. CC	H-B	7	0.000	0.057	1.000	1.296 (0.992-1.691)	1.468 (1.353-1.592)
	TC+TT VS. CC	P-B	3	0.000	0.266	1.000	1.418 (0.767-2.621)	1.788 (1.542-2.072)
	TC+TT VS. CC	BC	5	0.000	0.116	1.000	1.367 (0.926-2.017)	1.596 (1.459-1.747)
	TT VS. CC	Overall	10	0.000	**0.011**	0.385	1.601 (1.112-2.305)	1.924 (1.674-2.210)
	TT VS. CC	Asian	7	0.000	**0.017**	0.595	1.684 (1.099-2.580)	1.969 (1.707-2.272)
	TT VS. CC	Caucasian	3	0.753	0.292	1.000	1.341 (0.757-2.376)	1.357 (0.770-2.391)
	TT VS. CC	MassArray	3	0.048	**0.000**	**0.000**	2.836 (1.981-4.059)	2.676 (2.217-3.230)
	TT VS. CC	RT-PCR	3	0.011	0.334	1.000	1.307 (0.759-2.249)	1.394 (1.093-1.776)
	TT VS. CC	TaqMan	4	0.226	0.784	1.000	0.964 (0.559-1.662)	0.943 (0.620-1.435)
	TT VS. CC	H-B	7	0.000	0.082	1.000	1.427 (0.956-2.129)	1.725 (1.479-2.012)
	TT VS. CC	P-B	3	0.024	0.064	1.000	2.167 (0.955-4.920)	3.043 (2.183-4.240)
	TT VS. CC	BC	5	0.000	0.102	1.000	1.674 (0.902-3.105)	2.088 (1.745-2.499)
	TT VS. TC+CC	Overall	10	0.001	**0.009**	0.315	1.453 (1.097-1.923)	1.619 (1.414-1.853)
	TT VS. TC+CC	Asian	7	0.000	**0.018**	0.630	1.482 (1.069-2.054)	1.639 (1.426-1.884)
	TT VS. TC+CC	Caucasian	3	0.744	0.328	1.000	1.309 (0.741-2.311)	1.325 (0.754-2.327)
	TT VS. TC+CC	MassArray	3	0.124	**0.000**	**0.000**	2.137 (1.606-2.843)	2.062 (1.719-2.474)
	TT VS. TC+CC	RT-PCR	3	0.166	**0.043**	1.000	1.256 (0.903-1.747)	1.276 (1.007-1.617)
	TT VS. TC+CC	TaqMan	4	0.225	0.684	1.000	0.941 (0.549-1.615)	0.918 (0.607-1.387)
	TT VS. TC+CC	H-B	7	0.005	0.076	1.000	1.314 (0.971-1.777)	1.477 (1.271-1.715)
	TT VS. TC+CC	P-B	3	0.154	**0.000**	**0.000**	2.030 (1.163-3.545)	2.397 (1.732-3.317)
	TT VS. TC+CC	BC	5	0.002	0.071	1.000	1.529 (0.965-2.424)	1.741 (1.461-2.075)
rs10836347	T VS. C	Overall	6	0.804	0.155	1.000	0.920 (0.818-1.033)	0.919 (0.818-1.033)
C > T	T VS. C	MassArray	3	0.391	0.318	1.000	0.925 (0.794-1.078)	0.925 (0.794-1.078)
	T VS. C	RT-PCR	3	0.811	0.308	1.000	0.912 (0.761-1.092)	0.911 (0.761-1.090)
	T VS. C	H-B	5	0.871	0.448	1.000	0.951 (0.833-1.086)	0.950 (0.832-1.084)
	TC VS. CC	Overall	6	0.828	0.346	1.000	0.942 (0.831-1.068)	0.942 (0.831-1.067)
	TC VS. CC	MassArray	3	0.370	0.370	1.000	0.928 (0.788-1.093)	0.928 (0.788-1.093)
	TC VS. CC	RT-PCR	3	0.959	0.692	1.000	0.961 (0.791-1.169)	0.961 (0.791-1.169)
	TC VS. CC	H-B	5	0.975	0.835	1.000	0.985 (0.854-1.136)	0.985 (0.854-1.136)
	TC+TT VS. CC	Overall	6	0.816	0.232	1.000	0.928 (0.820-1.050)	0.928 (0.820-1.049)
	TC+TT VS. CC	MassArray	3	0.366	0.336	1.000	0.924 (0.786-1.086)	0.924 (0.786-1.085)
	TC+TT VS. CC	RT-PCR	3	0.895	0.476	1.000	0.933 (0.771-1.130)	0.933 (0.771-1.129)
	TC+TT VS. CC	H-B	5	0.935	0.629	1.000	0.966 (0.840-1.112)	0.966 (0.840-1.111)
	TT VS. CC	Overall	6	0.973	0.171	1.000	0.683 (0.378-1.236)	0.664 (0.370-1.193)
	TT VS. CC	MassArray	3	0.937	0.618	1.000	0.803 (0.337-1.914)	0.802 (0.337-1.908)
	TT VS. CC	RT-PCR	3	0.785	0.168	1.000	0.594 (0.264-1.336)	0.570 (0.257-1.266)
	TT VS. CC	H-B	5	0.933	0.183	1.000	0.671 (0.352-1.279)	0.649 (0.344-1.226)
	TT VS. TC+CC	Overall	6	0.974	0.178	1.000	0.688 (0.380-1.244)	0.669 (0.373-1.200)
	TT VS. TC+CC	MassArray	3	0.944	0.633	1.000	0.810 (0.340-1.931)	0.810 (0.340-1.925)
	TT VS. TC+CC	RT-PCR	3	0.789	0.171	1.000	0.597 (0.265-1.341)	0.573 (0.258-1.272)
	TT VS. TC+CC	H-B	5	0.937	0.186	1.000	0.672 (0.353-1.281)	0.651 (0.345-1.229)
rs11821102	G VS. A	Overall	7	0.537	0.152	1.000	0.922 (0.825-1.030)	0.922 (0.825-1.030)
A > G	G VS. A	MassArray	3	0.655	0.163	1.000	0.896 (0.768-1.045)	0.896 (0.768-1.045)
	G VS. A	RT-PCR	3	0.633	0.216	1.000	0.896 (0.753-1.066)	0.896 (0.753-1.066)
	G VS. A	H-B	6	0.497	0.359	1.000	0.944 (0.834-1.068)	0.944 (0.834-1.068)
	G VS. A	Y	6	0.410	0.206	1.000	0.922 (0.812-1.047)	0.922 (0.813-1.046)
	GA VS. AA	Overall	7	0.483	0.116	1.000	0.906 (0.801-1.025)	0.906 (0.801-1.025)
	GA VS. AA	MassArray	3	0.719	0.080	1.000	0.859 (0.725-1.018)	0.859 (0.725-1.018)
	GA VS. AA	RT-PCR	3	0.469	0.306	1.000	0.904 (0.745-1.097)	0.904 (0.744-1.097)
	GA VS. AA	H-B	6	0.521	0.365	1.000	0.939 (0.818-1.077)	0.939 (0.818-1.076)
	GA VS. AA	Y	6	0.369	0.126	1.000	0.900 (0.778-1.041)	0.897 (0.781-1.031)
	GA+GG VS. AA	Overall	7	0.509	0.126	1.000	0.911 (0.809-1.027)	0.911 (0.809-1.027)
	GA+GG VS. AA	MassArray	3	0.723	0.109	1.000	0.874 (0.741-1.031)	0.874 (0.741-1.030)
	GA+GG VS. AA	RT-PCR	3	0.538	0.251	1.000	0.896 (0.743-1.081)	0.896 (0.743-1.081)
	GA+GG VS. AA	H-B	6	0.510	0.354	1.000	0.939 (0.822-1.072)	0.939 (0.822-1.072)
	GA+GG VS. AA	Y	6	0.386	0.154	1.000	0.908 (0.790-1.043)	0.907 (0.793-1.037)
	GG VS. AA	Overall	7	0.814	0.929	1.000	0.999 (0.656-1.523)	0.981 (0.649-1.483)
	GG VS. AA	MassArray	3	0.371	0.743	1.000	1.143 (0.626-2.090)	1.103 (0.613-1.986)
	GG VS. AA	RT-PCR	3	0.928	0.515	1.000	0.819 (0.439-1.528)	0.814 (0.438-1.514)
	GG VS. AA	H-B	6	0.719	0.818	1.000	0.968 (0.601-1.558)	0.947 (0.595-1.507)
	GG VS. AA	Y	6	0.757	0.836	1.000	1.085 (0.655-1.798)	1.053 (0.644-1.723)
	GG VS. GA+AA	Overall	7	0.815	0.976	1.000	1.013 (0.665-1.543)	0.994 (0.658-1.502)
	GG VS. GA+AA	MassArray	3	0.370	0.693	1.000	1.167 (0.639-2.132)	1.126 (0.626-2.025)
	GG VS. GA+AA	RT-PCR	3	0.916	0.540	1.000	0.829 (0.445-1.547)	0.824 (0.443-1.531)
	GG VS. GA+AA	H-B	6	0.724	0.844	1.000	0.976 (0.606-1.570)	0.954 (0.600-1.519)
	GG VS. GA+AA	Y	6	0.761	0.790	1.000	1.102 (0.665-1.825)	1.069 (0.654-1.747)
rs1425802	G VS. A	Overall	6	0.738	0.239	1.000	1.040 (0.974-1.111)	1.040 (0.974-1.110)
A > G	G VS. A	MassArray	3	0.900	0.868	1.000	1.007 (0.930-1.090)	1.007 (0.930-1.090)
	G VS. A	RT-PCR	2	0.788	0.195	1.000	1.092 (0.956-1.247)	1.092 (0.956-1.247)
	G VS. A	H-B	5	0.619	0.237	1.000	1.047 (0.970-1.129)	1.047 (0.970-1.129)
	G VS. A	N	2	0.788	0.195	1.000	1.092 (0.956-1.247)	1.092 (0.956-1.247)
	G VS. A	Y	4	0.571	0.536	1.000	1.024 (0.950-1.104)	1.024 (0.950-1.104)
	GA VS. AA	Overall	6	0.578	0.943	1.000	1.004 (0.902-1.117)	1.004 (0.902-1.117)
	GA VS. AA	MassArray	3	0.192	0.836	1.000	1.001 (0.843-1.188)	1.014 (0.891-1.154)
	GA VS. AA	RT-PCR	2	0.524	0.946	1.000	0.993 (0.798-1.234)	0.993 (0.798-1.234)
	GA VS. AA	H-B	5	0.693	0.566	1.000	0.964 (0.852-1.091)	0.964 (0.852-1.091)
	GA VS. AA	N	2	0.524	0.946	1.000	0.993 (0.798-1.234)	0.993 (0.798-1.234)
	GA VS. AA	Y	4	0.336	0.905	1.000	1.003 (0.879-1.145)	1.007 (0.892-1.139)
	GA+GG VS. AA	Overall	6	0.786	0.548	1.000	1.031 (0.933-1.139)	1.031 (0.934-1.139)
	GA+GG VS. AA	MassArray	3	0.352	0.837	1.000	1.012 (0.893-1.147)	1.013 (0.897-1.144)
	GA+GG VS. AA	RT-PCR	2	0.792	0.575	1.000	1.058 (0.869-1.289)	1.058 (0.868-1.289)
	GA+GG VS. AA	H-B	5	0.740	0.857	1.000	1.011 (0.901-1.134)	1.011 (0.901-1.134)
	GA+GG VS. AA	N	2	0.792	0.575	1.000	1.058 (0.869-1.289)	1.058 (0.868-1.289)
	GA+GG VS. AA	Y	4	0.517	0.712	1.000	1.022 (0.911-1.146)	1.022 (0.911-1.146)
	GG VS. AA	Overall	6	0.724	0.223	1.000	1.083 (0.953-1.231)	1.083 (0.953-1.231)
	GG VS. AA	MassArray	3	0.971	0.896	1.000	1.011 (0.862-1.185)	1.011 (0.862-1.185)
	GG VS. AA	RT-PCR	2	0.760	0.200	1.000	1.174 (0.919-1.501)	1.174 (0.919-1.501)
	GG VS. AA	H-B	5	0.632	0.187	1.000	1.104 (0.953-1.280)	1.104 (0.953-1.280)
	GG VS. AA	N	2	0.760	0.200	1.000	1.174 (0.919-1.501)	1.174 (0.919-1.501)
	GG VS. AA	Y	4	0.536	0.518	1.000	1.050 (0.904-1.221)	1.051 (0.904-1.221)
	GG VS. GA+AA	Overall	6	0.391	0.184	1.000	1.081 (0.964-1.212)	1.078 (0.965-1.206)
	GG VS. GA+AA	MassArray	3	0.706	0.956	1.000	1.004 (0.875-1.152)	1.004 (0.875-1.152)
	GG VS. GA+AA	RT-PCR	2	0.484	0.135	1.000	1.180 (0.952-1.464)	1.179 (0.950-1.463)
	GG VS. GA+AA	H-B	5	0.478	0.071	1.000	1.126 (0.990-1.280)	1.125 (0.990-1.280)
	GG VS. GA+AA	N	2	0.484	0.135	1.000	1.180 (0.952-1.464)	1.179 (0.950-1.463)
	GG VS. GA+AA	Y	4	0.284	0.515	1.000	1.055 (0.907-1.228)	1.044 (0.917-1.189)
rs187115	G VS. A	Overall	6	0.000	0.127	1.000	1.270 (0.935-1.725)	1.332 (1.198-1.481)
A > G	G VS. A	Asian	4	0.000	0.147	1.000	1.380 (0.893-2.134)	1.491 (1.310-1.697)
	G VS. A	Caucasian	2	0.899	0.458	1.000	1.071 (0.893-1.285)	1.071 (0.893-1.285)
	G VS. A	RT-PCR	2	0.580	**0.000**	**0.000**	1.762 (1.490-2.083)	1.765 (1.493-2.087)
	G VS. A	TaqMan	3	0.000	0.769	1.000	1.084 (0.633-1.855)	1.120 (0.958-1.309)
	G VS. A	H-B	5	0.000	0.148	1.000	1.310 (0.909-1.889)	1.405 (1.248-1.581)
	G VS. A	Y	5	0.000	0.148	1.000	1.310 (0.909-1.889)	1.405 (1.248-1.581)
	G VS. A	GBC	2	0.899	0.458	1.000	1.071 (0.893-1.285)	1.071 (0.893-1.285)
	GA VS. AA	Overall	6	0.000	0.130	1.000	1.276 (0.931-1.749)	1.349 (1.179-1.542)
	GA VS. AA	Asian	4	0.000	0.141	1.000	1.391 (0.897-2.158)	1.487 (1.269-1.743)
	GA VS. AA	Caucasian	2	0.885	0.633	1.000	1.063 (0.828-1.364)	1.063 (0.828-1.364)
	GA VS. AA	RT-PCR	2	0.301	**0.000**	**0.000**	1.756 (1.416-2.179)	1.765 (1.436-2.168)
	GA VS. AA	TaqMan	3	0.001	0.712	1.000	1.109 (0.642-1.915)	1.129 (0.923-1.380)
	GA VS. AA	H-B	5	0.000	0.147	1.000	1.317 (0.908-1.909)	1.407 (1.216-1.629)
	GA VS. AA	Y	5	0.000	0.147	1.000	1.317 (0.908-1.909)	1.407 (1.216-1.629)
	GA VS. AA	GBC	2	0.885	0.633	1.000	1.063 (0.828-1.364)	1.063 (0.828-1.364)
	GA+GG VS. AA	Overall	6	0.000	0.128	1.000	1.306 (0.926-1.843)	1.383 (1.219-1.570)
	GA+GG VS. AA	Asian	4	0.000	0.143	1.000	1.436 (0.884-2.331)	1.550 (1.331-1.805)
	GA+GG VS. AA	Caucasian	2	0.883	0.527	1.000	1.076 (0.857-1.351)	1.076 (0.857-1.351)
	GA+GG VS. AA	RT-PCR	2	0.383	**0.000**	**0.000**	1.872 (1.536-2.282)	1.876 (1.540-2.285)
	GA+GG VS. AA	TaqMan	3	0.000	0.733	1.000	1.110 (0.610-2.018)	1.138 (0.943-1.374)
	GA+GG VS. AA	H-B	5	0.000	0.147	1.000	1.353 (0.899-2.035)	1.457 (1.267-1.675)
	GA+GG VS. AA	Y	5	0.000	0.147	1.000	1.353 (0.899-2.035)	1.457 (1.267-1.675)
	GA+GG VS. AA	GBC	2	0.883	0.527	1.000	1.076 (0.857-1.351)	1.076 (0.857-1.351)
	GG VS. AA	Overall	6	0.002	0.113	1.000	1.580 (0.897-2.785)	1.562 (1.195-2.042)
	GG VS. AA	Asian	4	0.007	0.130	1.000	1.889 (0.830-4.299)	2.181 (1.502-3.169)
	GG VS. AA	Caucasian	2	0.995	0.565	1.000	1.118 (0.765-1.634)	1.118 (0.765-1.634)
	GG VS. AA	RT-PCR	2	0.903	**0.000**	**0.000**	2.949 (1.827-4.762)	2.940 (1.815-4.763)
	GG VS. AA	TaqMan	3	0.005	0.890	1.000	1.076 (0.380-3.046)	1.189 (0.821-1.722)
	GG VS. AA	H-B	5	0.003	0.133	1.000	1.699 (0.851-3.392)	1.849 (1.333-2.566)
	GG VS. AA	Y	5	0.003	0.133	1.000	1.699 (0.851-3.392)	1.849 (1.333-2.566)
	GG VS. AA	GBC	2	0.995	0.565	1.000	1.118 (0.765-1.634)	1.118 (0.765-1.634)
	GG VS. GA+AA	Overall	6	0.012	0.120	1.000	1.470 (0.905-2.388)	1.439 (1.105-1.873)
	GG VS. GA+AA	Asian	4	0.023	0.142	1.000	1.718 (0.835-3.533)	1.907 (1.315-2.765)
	GG VS. GA+AA	Caucasian	2	0.982	0.633	1.000	1.094 (0.756-1.585)	1.094 (0.756-1.585)
	GG VS. GA+AA	RT-PCR	2	0.764	**0.000**	**0.000**	2.470 (1.537-3.968)	2.452 (1.520-3.956)
	GG VS. GA+AA	TaqMan	3	0.019	0.908	1.000	1.054 (0.431-2.578)	1.140 (0.793-1.640)
	GG VS. GA+AA	H-B	5	0.017	0.137	1.000	1.572 (0.866-2.855)	1.663 (1.202-2.302)
	GG VS. GA+AA	Y	5	0.017	0.137	1.000	1.572 (0.866-2.855)	1.663 (1.202-2.302)
	GG VS. GA+AA	GBC	2	0.982	0.633	1.000	1.094 (0.756-1.585)	1.094 (0.756-1.585)
rs353639	C VS. A	Overall	4	0.192	0.280	1.000	1.077 (0.903-1.285)	1.080 (0.939-1.243)
A > C	C VS. A	Caucasian	3	0.377	0.897	1.000	1.009 (0.859-1.186)	1.011 (0.861-1.187)
	C VS. A	TaqMan	3	0.377	0.897	1.000	1.009 (0.859-1.186)	1.011 (0.861-1.187)
	C VS. A	H-B	2	0.068	0.593	1.000	1.106 (0.765-1.598)	1.107 (0.904-1.356)
	C VS. A	P-B	2	0.253	0.578	1.000	1.052 (0.841-1.315)	1.057 (0.870-1.283)
	C VS. A	Y	3	0.119	0.598	1.000	1.044 (0.818-1.332)	1.046 (0.885-1.236)
	C VS. A	GBC	2	0.221	0.633	1.000	1.042 (0.823-1.319)	1.048 (0.865-1.270)
	CA VS. AA	Overall	4	0.430	0.442	1.000	1.071 (0.899-1.276)	1.071 (0.899-1.275)
	CA VS. AA	Caucasian	3	0.718	0.924	1.000	0.990 (0.806-1.215)	0.990 (0.807-1.215)
	CA VS. AA	TaqMan	3	0.718	0.924	1.000	0.990 (0.806-1.215)	0.990 (0.807-1.215)
	CA VS. AA	H-B	2	0.135	0.396	1.000	1.103 (0.762-1.596)	1.113 (0.869-1.425)
	CA VS. AA	P-B	2	0.559	0.812	1.000	1.030 (0.804-1.320)	1.030 (0.805-1.319)
	CA VS. AA	Y	3	0.256	0.580	1.000	1.056 (0.831-1.341)	1.060 (0.863-1.301)
	CA VS. AA	GBC	2	0.441	0.945	1.000	1.008 (0.788-1.290)	1.009 (0.789-1.290)
	CA+CC VS. AA	Overall	4	0.280	0.333	1.000	1.082 (0.897-1.307)	1.085 (0.919-1.282)
	CA+CC VS. AA	Caucasian	3	0.524	0.987	1.000	1.001 (0.825-1.215)	1.002 (0.825-1.216)
	CA+CC VS. AA	TaqMan	3	0.524	0.987	1.000	1.001 (0.825-1.215)	1.002 (0.825-1.216)
	CA+CC VS. AA	H-B	2	0.089	0.604	1.000	1.113 (0.743-1.666)	1.123 (0.886-1.423)
	CA+CC VS. AA	P-B	2	0.377	0.680	1.000	1.050 (0.831-1.326)	1.050 (0.832-1.326)
	CA+CC VS. AA	Y	3	0.162	0.562	1.000	1.053 (0.807-1.374)	1.060 (0.871-1.291)
	CA+CC VS. AA	GBC	2	0.303	0.781	1.000	1.032 (0.813-1.310)	1.033 (0.820-1.302)
	CC VS. AA	Overall	4	0.467	0.456	1.000	1.147 (0.788-1.668)	1.152 (0.794-1.672)
	CC VS. AA	Caucasian	3	0.492	0.780	1.000	1.050 (0.698-1.580)	1.059 (0.707-1.586)
	CC VS. AA	TaqMan	3	0.492	0.780	1.000	1.050 (0.698-1.580)	1.059 (0.707-1.586)
	CC VS. AA	H-B	2	0.222	0.628	1.000	1.188 (0.577-2.448)	1.155 (0.644-2.072)
	CC VS. AA	P-B	2	0.305	0.571	1.000	1.138 (0.686-1.886)	1.150 (0.709-1.864)
	CC VS. AA	Y	3	0.372	0.917	1.000	1.024 (0.636-1.650)	1.026 (0.637-1.650)
	CC VS. AA	GBC	2	0.345	0.550	1.000	1.143 (0.716-1.826)	1.153 (0.723-1.837)
	CC VS.CA+AA	Overall	4	0.572	0.490	1.000	1.134 (0.783-1.642)	1.138 (0.788-1.644)
	CC VS.CA+AA	Caucasian	3	0.561	0.776	1.000	1.052 (0.703-1.574)	1.060 (0.711-1.578)
	CC VS.CA+AA	TaqMan	3	0.561	0.776	1.000	1.052 (0.703-1.574)	1.060 (0.711-1.578)
	CC VS.CA+AA	H-B	2	0.293	0.650	1.000	1.150 (0.626-2.112)	1.143 (0.642-2.036)
	CC VS.CA+AA	P-B	2	0.344	0.603	1.000	1.128 (0.695-1.832)	1.135 (0.704-1.829)
	CC VS.CA+AA	Y	3	0.466	0.920	1.000	1.023 (0.639-1.639)	1.024 (0.640-1.639)
	CC VS.CA+AA	GBC	2	0.408	0.559	1.000	1.139 (0.718-1.808)	1.147 (0.724-1.816)
rs713330	T VS. C	Overall	6	0.908	0.546	1.000	0.966 (0.865-1.080)	0.967 (0.865-1.080)
C > T	T VS. C	MassArray	3	0.892	0.620	1.000	0.967 (0.848-1.104)	0.967 (0.848-1.104)
	T VS. C	RT-PCR	2	0.661	0.409	1.000	0.905 (0.716-1.145)	0.906 (0.715-1.146)
	T VS. C	H-B	5	0.847	0.762	1.000	0.980 (0.860-1.116)	0.980 (0.861-1.116)
	TC VS. CC	Overall	6	0.874	0.772	1.000	0.982 (0.869-1.111)	0.982 (0.869-1.110)
	TC VS. CC	MassArray	3	0.958	0.488	1.000	0.949 (0.820-1.100)	0.949 (0.820-1.100)
	TC VS. CC	RT-PCR	2	0.428	0.889	1.000	1.020 (0.788-1.319)	1.019 (0.787-1.319)
	TC VS. CC	H-B	5	0.813	0.995	1.000	1.001 (0.867-1.156)	1.000 (0.866-1.155)
	TC+TT VS. CC	Overall	6	0.921	0.651	1.000	0.973 (0.863-1.096)	0.973 (0.863-1.096)
	TC+TT VS. CC	MassArray	3	0.953	0.539	1.000	0.956 (0.829-1.103)	0.956 (0.829-1.103)
	TC+TT VS. CC	RT-PCR	2	0.521	0.739	1.000	0.958 (0.745-1.233)	0.958 (0.744-1.233)
	TC+TT VS. CC	H-B	5	0.874	0.882	1.000	0.989 (0.860-1.139)	0.989 (0.860-1.139)
	TT VS. CC	Overall	6	0.514	0.484	1.000	0.892 (0.563-1.414)	0.853 (0.547-1.331)
	TT VS. CC	MassArray	3	0.526	0.807	1.000	1.059 (0.622-1.806)	1.068 (0.630-1.809)
	TT VS. CC	RT-PCR	2	0.500	0.065	1.000	0.394 (0.130-1.198)	0.357 (0.119-1.067)
	TT VS. CC	H-B	5	0.373	0.549	1.000	0.903 (0.506-1.613)	0.852 (0.505-1.438)
	TT VS. TC+CC	Overall	6	0.505	0.492	1.000	0.897 (0.566-1.421)	0.856 (0.549-1.335)
	TT VS. TC+CC	MassArray	3	0.523	0.781	1.000	1.069 (0.628-1.821)	1.078 (0.637-1.825)
	TT VS. TC+CC	RT-PCR	2	0.485	0.064	1.000	0.396 (0.130-1.201)	0.357 (0.119-1.064)
	TT VS. TC+CC	H-B	5	0.365	0.548	1.000	0.903 (0.502-1.624)	0.852 (0.505-1.437)

*P*H=*P*-value of heterogeneity test; *P*Z=*P*-value of Z test; BC: breast cancer; GBC: Gallbladder cancer; RT-PCR: reverse transcription-polymerase chain reaction; P-B: population-based; H-B: hospital-based; HWE: Hardy Weinberg Equilibrium; Y: studies were conformed to HWE; N: studies were not conformed to HWE.

### Subgroup analysis

Results of the subgroup analyses were also shown in Table 2. We performed stratified analyses according to source of control, ethnicity, genotyping method and HWE status. No significant association of rs13347 polymorphism and cancer risk was identified for Asian and Caucasian subgroups (Table 2). When the stratification analysis was conducted based on source of control, we uncovered that population-based (P-B) group was the source of heterogeneity in recessive model (TT vs. TC+CC: OR=2.397, 95%CI: 0.732-3.317, *P*_Adjust_ < 0.001) rather than hospital-based (H-B) group. Subsequently, we also conducted a subgroup analysis referring to genotyping method. In the MassArray group, statistical heterogeneity preserved significance in all the genetic models (T vs. C: OR = 1.766, 95%CI: 1.454-2.144, *P*_Adjust_ < 0.001; TC vs. CC: OR = 1.857, 95%CI: 1.528-2.257, *P*_Adjust_ < 0.001; TC+TT vs. CC: OR = 2.003, 95%CI: 1.603-2.502, *P*_Adjust_ < 0.001; TT vs. CC: OR = 2.836, 95%CI: 1.981-4.059, *P*_Adjust_ < 0.001; TT vs. TC+CC: OR = 2.062, 95%CI: 1.719-2.474, *P*_Adjust_ < 0.001). In contrast, no significant association between rs13347 polymorphism and cancer risk was identified for either the RT-PCR or the TaqMan groups (Table 2). Finally, when stratified by cancer type, we found no association between rs13347 polymorphism and BC risk (Table 2).

For the rs187115 polymorphism, a significantly increased association was observed in the RT-PCR group upon stratifying by genotyping For the rs187115 polymorphism, a significantly increased association was observed in the RT-PCR group upon stratifying by genotyping method, indicating RT-PCR group can account for the source of heterogeneity. (G vs. A: OR = 1.765, 95%CI: 1.493-2.087, *P*_Adjust_ < 0.001; GA vs. TT: OR = 1.765, 95%CI: 1.436-2.168, *P*_Adjust_ < 0.001; GA+GG vs. AA: OR = 1.876, 95%CI: 1.540-2.285, *P*_Adjust_ < 0.001; GG vs. TT: OR = 2.940, 95%CI: 1.815-4.763, *P*_Adjust_ < 0.001; GG vs. GA+AA: OR = 2.452, 95%CI: 1.520-3.956, *P*_Adjust_ < 0.001; Table 2). However, no association was found in the TaqMan group. Subgroup analysis based on ethnicity presented that rs187115 polymorphism was not related to cancer risk for both Asian and Caucasian populations (Table 2).

For the remaining *CD44* polymorphisms, when stratified analysis by genotyping method, source of control, ethnicity, cancer type and HWE status, no significant association was identified from the pooled results (Table 2).

### Sensitivity analysis and publication bias

Sensitivity analysis was conducted to evaluate the stability of pooled ORs, in which an individual study will be removed each time in turn from the pooled analyses to detect the influence of individual case-control studies on the pooled ORs. We identified that removal of any single case-control study did not influence the stability of the results. We also generated Egger's funnel plot and conducted Begg's test to assess the publication bias. The shapes of funnel plot appeared symmetrical, indicating no publication bias was existed. These findings were further supported by Egger's funnel plot for the seven *CD44* polymorphisms (rs1425802, rs10836347, rs11821102, rs13347, rs187115, rs353639 and rs713330) Table S1.

Additionally, PRISMA 2009 Checklist for this Meta-analysis was presented in Supplementary Table 2, and the quality of the enrolled studies was shown in Table 3, which was evaluated by Newcastle-Ottawa Scale (NOS).

**Table 3:** Methodological quality of the included studies according to the Newcastle-Ottawa Scale

Variants	Author	Ethnicity	Adequacy of Case Definition	Represent ativeness of the Cases	Selection of Controls	Definition of Controls	Comparability Cases/Controls	Ascertain ment of Exposure	Same Method of Ascertainment	Non- response rate	Total
rs10836347	Wu *et al.*	Asian	*	*	NA	*	**	*	*	*	8
C>T	Chou *et al.*	Asian	*	*	NA	*	**	*	*	*	8
	Wu *et al.*	Asian	*	*	NA	*	**	*	*	*	8
	Jiang *et al.*	Asian	*	*	NA	*	**	*	*	*	8
	Xiao *et al.*	Asian	*	*	*	*	**	*	*	*	9
	Chou *et al.*	Asian	*	*	NA	NA	**	*	*	*	7
rs11821102	Wu *et al.*	Asian	*	*	NA	*	**	*	*	*	8
A>G	Chou *et al.*	Asian	*	*	NA	*	**	*	*	*	8
	Wu *et al.*	Asian	*	*	NA	*	**	*	*	*	8
	Jiang *et al.*	Asian	*	*	NA	*	**	*	*	*	8
	Xiao *et al.*	Asian	*	*	*	*	**	*	*	*	9
	Chou *et al.*	Asian	*	*	NA	NA	**	*	*	*	7
rs13347	Wu *et al.*	Asian	*	*	NA	*	**	*	*	*	8
C>T	Chou *et al.*	Asian	*	*	NA	*	**	*	*	*	8
	Wu *et al.*	Asian	*	*	NA	*	**	*	*	*	8
	Tulsyan *et al.*	Caucasian	*	*	*	*	**	*	*	*	9
	Jiang *et al.*	Asian	*	*	NA	*	**	*	*	*	8
	Xiao *et al.*	Asian	*	*	*	*	**	*	*	*	9
	Chou *et al.*	Asian	*	*	NA	NA	**	*	*	*	7
	Sharma *et al.*	Caucasian	*	*	NA	*	**	*	*	*	8
	Yadav *et al.*	Caucasian	*	*	*	NA	**	*	*	*	8
rs1425802	Chou *et al.*	Asian	*	*	NA	*	**	*	*	*	8
A>G	Wu *et al.*	Asian	*	*	NA	*	**	*	*	*	8
	Jiang *et al.*	Asian	*	*	NA	*	**	*	*	*	8
	Xiao *et al.*	Asian	*	*	*	*	**	*	*	*	8
	Chou *et al.*	Asian	*	*	NA	NA	**	*	*	*	7
rs187115	Liu *et al.*	Asian	*	*	NA	*	**	*	*	*	8
A>G	Chou *et al.*	Asian	*	*	NA	*	**	*	*	*	8
	Sharma *et al.*	Caucasian	*	*	NA	*	**	*	*	*	8
	Chou *et al.*	Asian	*	*	NA	NA	**	*	*	*	7
	Yadav *et al.*	Caucasian	*	*	*	NA	**	*	*	*	8
rs353639	Tulsyan *et al.*	Caucasian	*	*	*	*	**	*	*	*	9
A>C	Sharma *et al.*	Caucasian	*	*	NA	*	**	*	*	*	8
	Qiu *et al.*	Asian	*	*	NA	*	**	*	*	*	8
	Yadav *et al.*	Caucasian	*	*	*	NA	**	*	*	*	8
rs713330	Chou *et al.*	Asian	*	*	NA	*	**	*	*	*	8
C>T	Wu *et al.*	Asian	*	*	NA	*	**	*	*	*	8
	Jiang *et al.*	Asian	*	*	NA	*	**	*	*	*	8
	Xiao *et al.*	Asian	*	*	*	*	**	*	*	*	9
	Chou *et al.*	Asian	*	*	NA	NA	**	*	*	*	7

This table identifies ‘high’ quality choices with a ‘star’. A study can be awarded a maximum of 1 star for each numbered item within the Selection and Exposure categories. A maximum of 2 stars can be given for Comparability. *, Yes; NA, not applicable. (http://www.ohri.ca/programs/clinical_epidemiology/oxford.htm).

### Linkage disequilibrium (LD) analysis across populations

In order to better understand these results, LD analysis was performed to test the existence of bins. However, only six polymorphisms could be matched from the database, including rs10836347, rs11821102, rs13347, rs187115, rs353639 and rs713330 polymorphisms. LD plots for the CEU population showed a moderate LD value (r^2^≥0.5) between rs187115 and rs353639 polymorphisms. Additionally, LD plots for the YRI population showed a moderate LD value (r^2^>0.6) between rs11821102 and rs13347 polymorphisms (Supplementary Figure S1).

## DISCUSSION

Currently, personalized analyses and improved methods for cancer diagnoses can be offered by preferable comprehending the association between genetic polymorphisms and malignancies risk. Among the polymorphisms widely researched for risk factors associated with cancers, *CD44* has become a common target gene.

CD44 is involved in many cellular processes, such as angiogenesis, proliferation, and metastasis [21]. The *CD44* is composed 20 exons grouped into two areas [22]. Group 1 is comprised of co-expressed exons 1-5 and 16-20, while group 2 is comprised of exons 6-15. Ten exons in group 1 are spliced alternatively (exons 5 and 16). Multi-functional characteristics of CD44 contribute to the binding of its ligand, hyaluronan [23]. Two binding domains are available for hyaluronan, encoded by exons 2 and 5[24]. Interaction of hyaluronan with CD44 facilitates the regulation of BC via cell to cell adhesion and suppressed invasion [25]. Alterations in binding of hyaluronan to CD44 leads to the activation of invasion and metastasis in BC [26, 27], sarcoma and GC [28, 29]. Based on these findings, we predicted that *CD44* would have a significant impact on the pathogenesis and prognosis of many cancer types.

A previous study performed by Tulsyan *et al*. [12] aimed to determine if genetic variants (rs13347 and rs353639) of *CD44* influence individuals' risk for BC in 258 cases and 131 healthy controls. However, no significant differences were addressed. Their results were not consistent with Jiang *et al.*'s work [9], in which the authors evaluated the rs13347 polymorphism in a Chinese population consisted of 1,853 BC patients and 1,992 healthy controls and identified that variant genotype (CT+TT) conferred a 1.72-fold increased risk of BC. In addition, they also carried out a reporter assay to verify these findings and elucidated that CT+TT genotype carriers have higher expression of CD44 than wildtype CC carriers. The differences in these findings can be attributed to the differences in ethnicities or the presence of another linked *CD44* polymorphism that confers risk in Chinese population. Another study conducted by Xiao *et al.*[18] reports that *CD44* rs13347 C > T polymorphism is a susceptibility factor for nasopharyngeal carcinoma (NPC). Subsequently, Sharma *et al.*[16] re-considered the role of four *CD44* polymorphisms (rs13347, rs353639, rs187116 and rs187115) and gall bladder cancer (GBC) risk, and they found no significant difference in the frequency distribution of selected polymorphisms in GBC cases when compared with controls at either allelic or genotypic levels in a North Indian population.

The conclusions from enrolled studies were controversial, and independent studies may not have sufficient statistical strength to precisely identify the effects of *CD44* polymorphisms on overall cancer risk. Thus, our team performed a quantitative meta-analysis to allow for increasing statistical power and provide multiple lines of evidence for the relationship between *CD44* polymorphisms and cancer risk. A total of 45 case-control studies were enrolled for the seven polymorphisms (rs10836347, rs11821102, rs13347, rs1425802, rs187115, and rs353639 and rs713330). Finally, we identified that the mutated B allele of *CD44* polymorphisms was not observed to be associated with an increased risk of cancer. Nevertheless, it is worth noting that our data was not consistent with previously published studies, including a meta-analysis. In a study by Weng *et al.*[20], the authors found that carriers of the *CD44* rs187115 polymorphism with the genotype of at least one G were at an increased risk of developing transitional cell carcinoma (TCC). Around the same time, a similar finding was obtained from a study by Chou *et al.*[14], which found that *CD44* rs187115 polymorphism may serve as a biomarker for predicting prognosis of late-stage hepatocellular carcinoma (HCC). Furthermore, a study by Xiao *et al.*[18] revealed a positive relationship between the *CD44* rs13347 (C > T) polymorphism and NPC development. When the data were stratified based on genotyping method, *CD44* rs13347 polymorphism was found to be associated with an increased risk of cancer in the MassArray group in all the five genetic models. Additionally, in the RT-PCR subgroup, we also observed a significant increased association between the rs187115 polymorphism and cancer risk in all the genetic models. Moreover, subgroup analysis based on source of control suggests that a significant association was existed between rs13347 polymorphism and cancer risk in recessive model in P-B group. The existence of this phenomenon may be due to the inconsistencies in control groups. Although most of the controls were chose from healthy populations, many individuals may have suffered from other non-cancer diseases. These differences in control case characteristics could make our findings biased. On the other hand, we also observed significant between-study heterogeneity in our analysis. Absolute meta-regression analysis revealed that the genotyping method introduced substantial heterogeneity. Methodological problems are reflected in the deviations in HWE status, such as the errors in genotyping, the bias of population stratification or selection. Although we did not exclude these studies that were deviated from HWE, we have conducted a subgroup analysis by HWE status. We proved that HWE status did not give rise to bias of results. In addition, the stability of these results were further enhanced by sensitivity analysis.

The current meta-analysis comes with some advantages. Firstly, we have conducted a comprehensive search to identify more eligible studies thus, makes our analysis more persuasive and substantial. Secondly, quality of enrolled studies were all assessed by Newcastle-Ottawa Scale (NOS), so low quality studies should be excluded in order to raise the overall quality. Thirdly, subgroup analysis was performed according to cancer type, HWE status and so on at the aim of further deeply exploring the sources of heterogeneity. Fourthly, results were adjusted according to the recognized formula, ensuring the accuracy of the results. In addition, the stability of these studies was further confirmed by sensitivity analysis, and publication bias was tested by Egger's test and Begg's funnel plot. Finally, we have carefully searched for the databases and identified one recent published meta-analysis, which conducted by Shi and his colleagues [30]. They payed attention to the association of *CD44* rs13347 genetic polymorphism and cancer risk, and their ethnicity was restricted to Asians [30]. However, in our study, we analyzed seven polymorphisms in *CD44* and cancer risk, and the ethnicity comprised Asians and Caucasians. The largely increased sample size of current work provides us with more sufficient power to identify some conceal findings. In the end, Shi *et al.*[30] suggested that *CD44* rs13347 (C>T) polymorphism was related to an increased risk of human cancer in Asian people, especially in Chinese populations. Different from their work, we observed the mutated B allele of all *CD44* polymorphisms was not associated with the risk of cancer after adjusting. However, several drawbacks in our study should also be noted. Firstly, a relatively small number of studies were enrolled for each polymorphism, with a particularly small number of studies analyzing for the rs353639 polymorphism (only four case-control studies). This limitation may have resulted in an insufficient power for identifying minor association between *CD44* polymorphisms and cancer risk. Secondly, further studies are warranted to evaluate the effects of *CD44* polymorphisms on cancer risk in different ethnicities. In ethnicity subgroup analysis, the enrolled studies were restricted to Asian and Caucasian populations; data for other ethnicities were not analyzed. Thirdly, the phenotype of our study was a heterogeneous aggregation of a variety of cancer types, and only for part of *CD44* polymorphisms, a subgroup analysis based on cancer type was conducted, while for others, attributing to the limited number of studies for specific cancers, such as BC and CC, we were unable to validate the potential effects on these cancers homogeneous or not, which should be investigated in the future. Additionally, several potentially confounding factors were not considered in this study, such as age, sex, smoking and drinking status, (hepatitis B virus) HBV/ (hepatitis C virus) HCV carrier status, environmental factors, and so on.

## CONCLUSIONS

Our meta-analysis suggests that *CD44* polymorphisms might not represent risk factors for cancer. However, our findings require further validation in more well-designed studies with larger sample sizes in order to strengthen our conclusions.

## MATERIALS AND METHODS

### Search Strategy

We carried out a comprehensive literature search on Embase, Cochrane Library and PubMed (up to April 2, 2016) to find all relevant publications exploring the relationship between *CD44* polymorphisms and the risk of cancer. The search terms were as follows: “CD44” AND “SNP OR polymorphism OR mutation OR allele OR variation” AND “cancer OR adenocarcinoma OR carcinoma OR tumor OR neoplasm OR Leukemia OR lymphoma.” The language was restricted to English. These publications were extracted by two reviewers to identify studies specific to various cancers. We then carried out a manual retrieve of the references lists of these enrolled original publications/Reviews to identify additional eligible case-control studies.

### Inclusion and Exclusion Criteria

Enrolled studies should meet the following criteria: 1) they should assess the association between *CD44* polymorphisms and cancer risk; 2) they should be case-control/cohort studies; and 3) they should comprise sufficient data (allele and genotype frequencies). In addition, studies were excluded when they were: 1) case only studies, such as Reviews/comments/case reports and 2) not containing sufficient data.

### Data Extraction

Two reviewers (Meng Zhang and Yangyang Wang) performed the data extraction process based on the previously described enrollment criteria. All discrepancies were discussed until consensuses were obtained. In addition, the following characteristics were also extracted from publications: name of first author, publication year, ethnicity of the subjects in the case-control study, source of control, genotype frequency, and etc.

### Statistical analysis

ORs correspondence with 95%CIs were calculated to evaluate the strength of the relationship between *CD44* polymorphisms and cancer risk in five genetic models: allele contrast (B vs. A), dominant (BB + BA vs. AA), recessive (BB vs. BA + AA), homozygous (BB vs. AA), and heterozygous (BA vs. AA) models (A: wild type allele; B: variant allele). Subsequently, stratified analyses were performed by cancer type, ethnicity, source of control and genotyping method. We evaluated the statistical heterogeneity assumption by I^2^ statistics to quantify any inconsistency arising from inter-research variability derived from heterogeneity instead of random chance. An I^2^ value more than 50% was regarded as significant heterogeneity among these studies. In that case, pooled OR estimations of individual studies were tested by random effect model; if not, fixed effect model will be employed. Moreover, sensitivity analysis was carried out to verify the stability of our results and Egger's regression test and Begg's funnel plot were carried out to evaluate the publication bias. STATA 12.0 software was employed to calculate all the statistical analyses (STATA Corp, College Station, TX). In addition, Bonferroni corrections were also performed to adjust the results [31]. *P*<0.05 was regarded as statistically significant. Besides, this study is a systemic review of the literature, so ethical approval was not required.

### LD analysis across populations

Data was extracted from the International HapMap Project (http://hapmap.ncbi.nlm.nih.gov/cgi-perl/gbrowse/hapmap24_B36/#search), which comprises *CD44* polymorphisms evaluated in the current study. Briefly, populations incorporated in the project including YRI (Yoruba in Ibadan, Nigeria), CHB (Han Chinese in Beijing, China), JPT (Japanese in Tokyo, Japan) and CEU (Utah residents with northern and western Europe ancestry). Then, Haploview software was employed to conduct the analysis and LD was evaluated by r^2^ statistics in each of the populations [32].

## SUPPLEMENTARY FIGURE AND TABLES






