# Supplementary material for: Common polymorphisms in CD44 gene and susceptibility to cancer: a systematic review and meta-analysis of 45 studies
Source: Oncotarget. 2016 Oct 12;7(46):76021–35. doi: 10.18632/oncotarget.12580 (PMC5342795; doi:10.18632/oncotarget.12580)
Supplement: Supplementary file 1 [file oncotarget-07-76021-s001.pdf]

Common polymorphisms in *CD44* gene and susceptibility to cancer: A systematic review and meta-analysis of 45 studies

SUPPLEMENTARY FIGURE AND TABLES

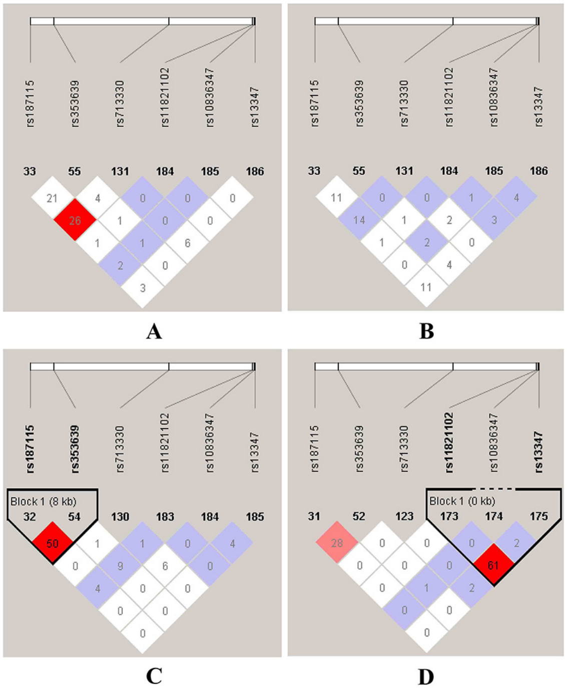

Supplementary Figure S1: Linkage disequilibrium plot. The number of each cell represents r2 and white color cells shows no LD between polymorphisms. **A.** CHB (Han Chinese in Beijing, China); **B.** JPT (Japanese in Tokyo, Japan); **C:** CEU (Utah residents with ancestry from northern and western Europe); **D:** YRI (Yoruba in Ibadan, Nigeria). The "rs" numbers are SNP IDs taken from National Center for Biotechnology Information (NCBI).

Supplementary Table S1: Egger's regression test for each polymorphism.

See Supplementary File 1

Supplementary Table S2: PRISMA 2009 Checklist For this Meta-analysis.

See Supplementary File 2
