# Supplementary material for: Common polymorphisms in CD44 gene and susceptibility to cancer: a systematic review and meta-analysis of 45 studies
Source: Oncotarget. 2016 Oct 12;7(46):76021–35. doi: 10.18632/oncotarget.12580 (PMC5342795; doi:10.18632/oncotarget.12580)
Supplement: Supplementary file 2 [file oncotarget-07-76021-s002.docx]

**Table S1.** Egger’s regression test for each polymorphism.

| **Polymorphism** | **Subgroup** | **Egger's test P>\|t\|** |
| --- | --- | --- |
|  |  |  |
| rs13347 | Overall | 0.097 |
|  | Asian | 0.217 |
|  | Caucasian | 0.798 |
|  | H-B | 0.170 |
|  | P-B | 0.147 |
|  | MassArray | 0.607 |
|  | RT-PCR | 0.538 |
|  | TaqMan | 0.678 |
|  | BC | 0.454 |
| rs10836347 | Overall | 0.896 |
|  | MassArray | 0.565 |
|  | RT-PCR | 0.480 |
|  | H-B | 0.812 |
| rs11821102 | Overall | 0.490 |
|  | MassArray | 0.614 |
|  | RT-PCR | 0.938 |
|  | H-B | 0.631 |
| rs1425802 | Overall | 0.141 |
|  | MassArray | 0.279 |
|  | H-B | 0.225 |
| rs187115 | Overall | 0.218 |
|  | Asian | 0.279 |
|  | H-B | 0.149 |
| rs713330 | Overall | 0.390 |
|  | MassArray | 0.572 |
|  | H-B | 0.570 |
| rs353639 | Overall | 0.522 |

H-B: hospital-based; P-B: population-based; RT-PCR: reverse transcription-polymerase chain reaction;
